# Evaluation of volume measurements of neuroanatomical structures related to speech in multiple sclerosis patients

**DOI:** 10.55730/1300-0144.5839

**Published:** 2024-07-16

**Authors:** Hıdır PEKMEZ, Anıl ALTIPARMAK, Feyza İNCEOĞLU, Mehmet AKÇİÇEK, Aslı BOLAYIR, Zeynep ÖZBAY, Merve AYDIN, Muhammed Furkan ARPACI

**Affiliations:** 1 Division of Anatomy, Department of Basic Medical Sciences, Faculty of Medicine, Malatya Turgut Özal University, Malatya, Turkiye; 2 Division of Anatomy, Department of Basic Medical Sciences, Institute of Graduate Science, Malatya Turgut Özal University, Malatya, Turkiye; 3 Division of Biostatistics, Department of Basic Medical Sciences, Faculty of Medicine, Malatya Turgut Özal University, Malatya, Turkiye; 4 Division of Radiology, Department of Internal Medicine, Faculty of Medicine, Malatya Turgut Özal University, Malatya, Turkiye; 5 Division of Neurology, Department of Internal Medicine, Faculty of Medicine, Malatya Turgut Özal University, Malatya, Turkiye

**Keywords:** Multiple sclerosis, speech, brain, magnetic resonance imaging, volume, VolBrain

## Abstract

**Background/aim:**

Individuals with multiple sclerosis (MS) may experience various speech-related issues, including decreased speech rate, increased pauses, and changes in speech rhythms. The purpose of this study was to compare the volumes of speech-related neuroanatomical structures in MS patients with those in a control group.

**Materials and methods:**

The research was conducted in the Neurology and Radiology Departments of Malatya Training and Research Hospital. The records of patients who presented to the Neurology Department between 2019 and 2022 were examined. The study included the magnetic resonance imaging (MRI) findings of 100 individuals, with 50 in the control group and 50 patients with MS, who had applied to the hospital in the specified years. VolBrain is a free system that works automatically over the internet (http://volbrain.upv.es/), enabling the measurement of brain volumes without human interaction. The acquired images were analyzed using the VolBrain program.

**Results:**

As a result of our research, a significant decrease was found in the volume of 18 of 26 speech-related regions in MS patients. It was determined that whole brain volumes decreased in the MS group compared to the control group.

**Conclusion:**

In our study, volume measurements of more speech-related areas were performed, unlike the few related studies previously conducted. We observed significant atrophy findings in the speech-related areas of the frontal, temporal, and parietal lobes of MS patients.

## 1. Introduction

Multiple sclerosis (MS) is a chronic illness characterized by inflammation in the central nervous system (CNS) [1,2]. This disease affects approximately 2.8 million people globally and is commonly diagnosed in individuals between the ages of 20 and 50 years. It is more frequently diagnosed in women than in men [3,4].

MS is characterized by clinical symptoms arising from lesions of the brain or spinal cord [1,2]. The signs and symptoms of MS vary depending on the location and size of the lesions in the CNS that lead to plaque formation. These differences are unique to each individual [5]. People who suffer from MS typically experience a variety of symptoms that can be quite debilitating. These symptoms may include feelings of exhaustion, physical discomfort, problems with controlling the bladder and bowels, difficulties with thinking and emotions, issues with seeing clearly, and challenges with speaking and swallowing. Unfortunately, these symptoms can significantly interfere with the capacity to perform routine tasks and engage in normal daily activities [6,7].

Individuals with MS may encounter speech problems. Today, the dual-flow model proposed by Hickok and Poeppel is used to understand the functional neuroanatomy of speech [8]. Motor and sensory areas in the dual-flow model include the gyrus precentralis, gyrus frontalis superior, gyrus postcentralis, gyrus frontalis inferior, and gyrus temporalis superior areas as a highly complex brain network also consisting of the lobulus parietalis inferior and gyrus temporalis medius structures, which are responsible for language functions and the auditory processing of speech [9].

People with MS may experience communication difficulties, especially with speech. This can negatively impact their ability to participate in conversations [10]. Speech disorders negatively affect social life and quality of life [11]. Speech is a delicate motor skill that demands accurate muscle coordination. It has been observed that 40% of individuals with MS experience dysarthria. Spastic, ataxic, and mixed spastic-ataxic dysarthria are prevalent clinical symptoms. The most common symptoms of dysarthria in MS are impaired voice control, rigidity, defective articulation and impaired emphasis, excessive loudness changes, and slow-paced speech. As the disease progresses, speech symptoms may become more severe [10,12]. Reproducible brain volume analysis techniques can measure and track brain atrophy over time [13].

The purpose of this study was to compare the volumes of speech-related neuroanatomical structures in MS patients with those in a control group.

## 2. Materials and methods

The research was conducted in the Neurology and Radiology Departments of Malatya Training and Research Hospital. The records of patients who presented to the Neurology Department between 2019 and 2022 were examined. Ethical approval was obtained with the decision of the Malatya Turgut Özal University Non-Invasive Clinical Research Ethics Committee numbered 2022/18-173 and dated 01.11.2022.

This study included the magnetic resonance imaging (MRI) scans of 100 individuals, with 50 in the control group and 50 patients with the relapsing-remitting MS subtype. There is no definitive clinical or laboratory diagnostic test for MS disease, but the McDonald diagnostic criteria are generally used today [14]. Therefore, diagnostic criteria entailed parameters obtained via clinical, laboratory, and imaging methods. Individuals with mental retardation, hemiplegia, diseases that could affect brain volume, or a history of head trauma or cranial surgery were not included in the study. The control group comprised healthy individuals who were admitted to the hospital for headache or dizziness and underwent MRI scans that showed no cranial pathology. These individuals did not have any illnesses that would affect their speech and had not experienced any events that would impact speech.

MRI was performed by taking axial T1-weighted images with an Amira 1.5-T device (Siemens, Erlangen, Germany). The MRI protocol was as follows: 3D T1-MPRAGE TR (repetition time), 2200 ms; TE (echo time), 2.79 ms; flip angle (declination), 8°; field of view, 250 mm; number of sections, 192; section thickness, 1 mm; matrix, 205 × 320. The acquired images were analyzed using the VolBrain program.

VolBrain is a free system that works automatically over the internet (http://volbrain.upv.es/), enabling the measurement of brain volumes without human interaction. It automatically performs volumetric brain analysis on T1-weighted images [15].

In our study, using VolBrain, the superior frontal gyrus and its medial segment, opercular inferior frontal gyrus, orbital inferior frontal gyrus, triangular inferior frontal gyrus, precentral gyrus, gyrus postcentralis, postcentral gyrus medial segment, superior parietal lobule, precuneus, gyrus temporalis superior, temporalis medius, and gyrus temporalis inferior volumes were analyzed.

### 2.1. Statistical analysis

The study’s sample size was determined via power analysis using the G*Power 3.1 program, which calculated the minimum sample size required to be 80, with a minimum of 40 individuals in each group [16].

Data analysis was conducted using IBM SPSS Statistics 25. The analysis included the calculation of descriptive statistics using metrics such as number, percentage, mean, standard deviation, median, and range. To compare independent groups, the Mann–Whitney U test was utilized.

## 3. Results

This study included the MRI results of 100 individuals, with 50 healthy individuals in the control group and 50 patients with MS, who applied to the hospital in the specified years.

No statistically significant difference was detected between the patient and control groups according to age or sex (p > 0.05, Table 1). The groups showed homogeneous distribution for age and sex.

No statistically significant relationship was found between disease duration and Expanded Disability Status Scale scores in the patient group (p > 0.05, Table 2).

Significant differences were discovered between the patient and control groups in bilateral gyrus frontalis superior, superior frontal gyrus medial segment, opercular inferior frontal gyrus, and gyrus precentralis volumes when comparing the groups in terms of the lobus frontalis (p < 0.05) (Table 3).

There were significant difference in the volumes of certain brain regions between the patient and control groups. The medial segment of the bilateral postcentral gyrus, the superior parietal lobule, the precuneus, and the right postcentral gyrus in the lobus parietalis displayed significant differences (p < 0.05). However, no significant difference was detected in the volume of the left postcentral gyrus between the two groups (p > 0.05) (Table 4).

A statistically significant difference was found between the patient and control groups in bilateral gyrus temporalis superior and right gyrus temporalis medius volumes in the lobus temporalis (p < 0.05). However, no statistically significant difference was found between the patient and control groups for left gyrus temporalis medius and bilateral gyrus temporalis inferior volumes (p > 0.05) (Table 5).

When the volumes of neuroanatomical structures in the right and left hemispheres were compared in the patient group, the opercular inferior frontal gyrus, precentral gyrus, and postcentral gyrus volumes showed statistically significant differences between hemispheres (p < 0.05) (Table 6).

In the control group, a comparison of the volumes of neuroanatomical structures in the right and left hemispheres revealed statistically significant differences between certain areas. Volume differences were observed in the opercular inferior frontal gyrus, triangular inferior frontal gyrus, postcentral gyrus, superior temporal gyrus, and inferior temporal gyrus (p < 0.05) (Table 7).

## 4. Discussion

Since brain volumes and neurological functions are linked, speech disorders caused by MS and the volumes of speech-related brain regions may be related.

Many individuals with MS experience cognitive impairments. They may also display language difficulties, such as dysarthria or reduced fluency. Dysarthric speech, a motor speech disorder, is frequently seen in MS patients due to damage to the central and peripheral nervous systems [17]. According to the literature, symptoms of MS may include speaking slowly, making explosive sounds without joining syllables, and improperly stressing certain syllables due to a lack of coordination of speech muscles [18]. Our study was carried out retrospectively based on MRI data and no tests were conducted to evaluate speech and language disorders in the patients.

Brain atrophy is one of the essential findings in MS disease. Brain volume decreases progressively in MS patients. Research has found a notable connection between atrophy and neurological functions. For instance, certain cognitive abilities, such as visual-spatial memory and verbal memory, are linked to the size of particular areas of the cortex [19]. Furthermore, studies have indicated that issues like fatigue, memory capacity, depression, anxiety, and muscle difficulties are linked to brain volume [20–23]. In our study, significant atrophy findings were observed in the speech-related areas of the frontal, temporal, and parietal lobes of MS patients.

Studies have shown that 45% of MS patients have speech disorders [11]. Individuals with MS may experience various speech-related issues, including a decrease in speech rate, increased pauses, and changes in speech rhythms. Additionally, weakness in tone of voice and difficulty initiating speech are common speech impairments associated with MS [24]. Previous studies reported aphasia-like symptoms such as difficulty in naming objects or remembering words, decreased verbal fluency, repetition of words, and impaired spelling [25].

Measurements of various regions in the brains of MS patients were examined in previous studies. Pagani et al. [26] reviewed the MRI images of 466 MS patients and 279 healthy controls. In that study, a significant level of atrophy was detected in the right superior frontal gyrus, bilateral gyrus precentralis, and pars orbitalis section of the inferior frontal gyrus in MS patients compared to the control group. Similarly, in our study, bilateral gyrus precentralis volume decreased and bilateral volume loss was observed in the gyrus frontalis superior. The volume loss in the pars orbitalis part of the inferior frontal gyrus was not significant. In addition, in our study, the opercular part of the inferior frontal gyrus was significant in volume in the MS group compared to the control group. Among the findings of Pagani et al. [26], while the gyrus postcentralis, gyrus temporalis superior, and inferior were atrophied bilaterally in MS patients, the gyrus temporalis medius was significantly reduced only in the right hemisphere. Similarly, bilateral gyrus temporalis superior and right gyrus temporalis medius volumes were decreased in our study. The gyrus postcentralis volume was significantly reduced only on the right side. The change in the gyrus temporalis inferior was not significant.

In our study, the volume losses observed in the frontal, precentral gyrus, and precuneus of MS patients are similar to previous findings in the literature [27–29]. In addition, some studies have reported a decrease in left gyrus temporalis volume in MS patients [27,30]. In our study, only a reduction in the volume of the gyrus temporalis superior from the left temporal region was observed. In addition, no significant difference was found between the right and left temporal gyrus in the MS group.

As a result of our research, a significant decrease was found in the volumes of 18 of 26 speech-related regions in MS patients. It was determined that whole brain volumes were decreased in the MS group compared to the control group. However, the volume of the right gyrus temporalis medius was increased. Some functions in brain regions may be more dominant on the right or left side. Studies show that the right hemisphere has various language functions, but when the right is surgically removed, the left hemisphere can undertake those tasks [31]. The left gyrus temporalis medius being affected due to MS may have caused a compensation mechanism to develop in the right gyrus temporalis medius, which is associated with similar tasks [32].

In this study, volume measurements of more speech-related areas were performed in comparison to the few related studies conducted previously. Our study’s findings will contribute to future research. While our results are noteworthy, future studies could involve larger sample sizes and broaden the research by exploring the variances among different types of multiple sclerosis. Moreover, conducting speech tests on patients and investigating the correlation between their performance and brain volume could yield valuable insights.

## Figures and Tables

**Table 1:** Comparison of groups according to the distribution of demographic variables.

Variable	Group	n / %	Groups		Total	p
			MS patients	Control		
Sex	Male	n	19	19	38	0.582^a^
		%	38.0%	38.0%	38.0%	
	Female	n	31	31	62	
		%	62.0%	62.0%	62.0%	
Total		n	50	50	100	
		%	100.0%	100.0%	100.0%	
Variable	n	Group	Mean ± SD	M (range)		p
Age	50	MS patients	39.46 ± 12.35	38.5 (18–70)		0.715^b^
	50	Control	38.70 ± 13.98	37 (18–67)		

n: Number of samples;

a chi-square test value (χ^2^); M: median; SD: standard deviation;

b Mann–Whitney U test; significance at p < 0.05.

**Table 2:** Relationship between Expanded Disability Status Scale (EDSS) scores and disease duration.

		EDSS	Disease duration (months)
EDSS	r	1.000	−0.076
	p	.	0.598

r: Spearman’s rank correlation coefficient.

**Table 3:** Comparison of volumes in the lobus frontalis between groups.

Variables	Groups	Mean ± SD	M (range)	Test	p
Right gyrus frontalis superior	MS patients	12.56 ± 2.23	12.66 (7.06–16.52)	769.000	0.001*
	Control	14.39 ± 2.48	13.99 (9.91–21.36)		
Left gyrus frontalis superior	MS patients	12.62 ± 2.64	12.35 (6.76–18.24)	876.000	0.010*
	Control	14.06 ± 2.45	13.62 (9.13–20.13)		
Right superior frontal gyrus medial segment	MS patients	5.68 ± 1.29	5.69 (3.05–8.71)	935.000	0.030*
	Control	6.37 ± 1.28	6.11 (3.96–9.26)		
Left superior frontal gyrus medial segment	MS patients	5.24 ± 1.12	5.32 (2.83–8.2)	818.500	0.003*
	Control	6.01 ± 1.23	5.86 (2.79–9.16)		
Right opercular inferior frontal gyrus	MS patients	2.94 ± 0.67	3.04 (1.47–4.54)	756.500	0.001*
	Control	3.4 ± 0.61	3.44 (2.3–5.05)		
Left opercular inferior frontal gyrus	MS patients	2.52 ± 0.57	2.5 (1.51–3.95)	822.000	0.003*
	Control	2.88 ± 0.59	2.78 (1.75–4.26)		
Right triangular inferior frontal gyrus	MS patients	2.7 ± 0.62	2.65 (1.25–4.22)	1071.500	0.218
	Control	2.9 ± 0.67	2.89 (1.61–4.6)		
Left triangular inferior frontal gyrus	MS patients	3.01 ± 0.76	2.58 (1.51–4.59)	1047.500	0.163
	Control	3.21 ± 0.72	3.16 (1.93–5.02)		
Right orbital inferior frontal gyrus	MS patients	0.99 ± 0.39	0.92 (0.21–2)	1199.000	0.725
	Control	1.02 ± 0.37	1.03 (0.26–2.01)		
Left orbital inferior frontal gyrus	MS patients	1.02 ± 0.37	1.01 (0.35–2.15)	1036.500	0.141
	Control	1.11 ± 0.34	1.08 (0.4–1.98)		
Right gyrus precentralis	MS patients	10.54 ± 1.92	10.57 (6.16–14.64)	705.000	0.001*
	Control	12.15 ± 1.9	11.82 (7.55–17.14)		
Left gyrus precentralis	MS patients	11.46 ± 1.95	11.38 (6.83–15.5)	874.500	0.010*
	Control	12.54 ± 2.55	12.44 (1.09–18.25)		

SD: Standard deviation; M: median; p: Mann–Whitney U test;

* statistical significance at p < 0.05.

**Table 4:** Comparison of volumes in the lobus parietalis between groups.

Variables	Groups	Mean ± SD	M (range)	Test	p
Right postcentral gyrus	MS patients	8.15 ± 1.29	8.29 (5.56–11.8)	851.500	0.006*
	Control	8.93 ± 1.31	8.76 (6.55–12.27)		
Left postcentral gyrus	MS patients	9.55 ± 1.67	9.75 (5.32–13.36)	1002.500	0.088
	Control	10.15 ± 1.35	10.02 (7.41–13.78)		
Right postcentral gyrus medial segment	MS patients	0.81 ± 0.19	0.8 (0.38–1.21)	622.500	0.000*
	Control	1.01 ± 0.23	0.99 (0.56–1.59)		
Left postcentral gyrus medial segment	MS patients	0.86 ± 0.25	0.82 (0.45–1.65)	930.500	0.028*
	Control	0.96 ± 0.25	0.99 (0.46–1.5)		
Right superior parietal lobule	MS patients	9.5 ± 2.63	10.15 (1.01–14.53)	932.000	0.028*
	Control	10.68 ± 1.63	10.58 (7.33–14.19)		
Left superior parietal lobule	MS patients	10.31 ± 1.73	10.42 (6.38–15.37)	955.000	0.042*
	Control	11.05 ± 1.62	11.02 (8.29–15.24)		
Right precuneus	MS patients	10.89 ± 1.68	11 (6.85–14.53)	897.000	0.015*
	Control	12.14 ± 2.51	12.01 (8.08–19.42)		
Left precuneus	MS patients	11 ± 1.86	10.87 (7.25–15.07)	961.500	0.047*
	Control	11.99 ± 2.26	11.42 (8.35–17.84)		

SD: Standard deviation; M: median; p: Mann–Whitney U test;

* statistical significance at p < 0.05.

**Table 5:** Comparison of volumes in the lobus temporalis between groups.

Variables	Groups	Mean ± SD	M (range)	Test	p
Right gyrus temporalis superior	MS patients	6.57 ± 1.16	6.51 (3.43–8.45)	930.000	0.027*
	Control	7.12 ± 1.18	7.31 (4.32–9.39)		
Left gyrus temporalis superior	MS patients	6.97 ± 1.25	6.92 (3.64–9.9)	847.000	0.005*
	Control	7.65 ± 1.96	7.82 (0.81–11.85)		
Right gyrus temporalis medius	MS patients	13.44 ± 6.87	12.7 (8.67–58.73)	876.000	0.010*
	Control	13.39 ± 2.06	13.81 (10.81–18.86)		
Left gyrus temporalis medius	MS patients	12.03 ± 2.44	11.48 (5.56–17.33)	1088.000	0.264
	Control	12.56 ± 2.16	12.08 (9.31–18.1)		
Right gyrus temporalis inferior	MS patients	9.61 ± 2.37	9.7 (1.18–13.97)	1187.000	0.664
	Control	9.93 ± 1.8	9.77 (7.14–15.53)		
Left gyrus temporalis inferior	MS patients	10.33 ± 2.45	10.6 (1.31–15.06)	1032.000	0.133
	Control	11.23 ± 2.15	10.75 (7.78–16.71)		

SD: Standard deviation; M: median; p: Mann–Whitney U test;

* statistical significance at p < 0.05.

**Table 6:** Comparison of the volumes of the lobus frontalis, lobus parietalis, and lobus temporalis in the right and left hemispheres in the patient group.

Variables	Groups	Mean ± SD	M (range)	Test	p
Superior frontal gyrus	Right	12.56 ± 2.23	12.66 (7.06–16.52)	1245.000	0.973
	Left	12.62 ± 2.64	12.35 (6.76–18.24)		
Superior frontal gyrus medial segment	Right	5.68 ± 1.29	5.69 (3.05–8.71)	979.500	0.062
	Left	5.24 ± 1.12	5.32 (2.83–8.2)		
Opercular inferior frontal gyrus	Right	2.94 ± 0.67	3.04 (1.47–4.54)	800.000	0.002*
	Left	2.52 ± 0.57	2.5 (1.51–3.95)		
Triangular inferior frontal gyrus	Right	2.7 ± 0.62	2.65 (1.25–4.22)	994.500	0.078
	Left	3.01 ± 0.76	2.58 (1.51–4.59)		
Orbital inferior frontal gyrus	Right	0.99 ± 0.39	0.92 (0.21–2)	1208.500	0.775
	Left	1.02 ± 0.37	1.01 (0.35–2.15)		
Precentral gyrus	Right	10.54 ± 1.92	10.57 (6.16–14.64)	913.000	0.020*
	Left	11.46 ± 1.95	11.38 (6.83–15.5)		
Postcentral gyrus	Right	8.15 ± 1.29	8.29 (5.56–11.8)	590.000	0.001*
	Left	9.55 ± 1.67	9.75 (5.32–13.36)		
Postcentral gyrus medial segment	Right	0.81 ± 0.19	0.8 (0.38–1.21)	1174.000	0.600
	Left	0.86 ± 0.25	0.82 (0.45–1.65)		
Superior parietal lobule	Right	9.5 ± 2.63	10.15 (1.01–14.53)	1012.000	0.101
	Left	10.31 ± 1.73	10.42 (6.38–15.37)		
Precuneus	Right	10.89 ± 1.68	11 (6.85–14.53)	1217.500	0.823
	Left	11 ± 1.86	10.87 (7.25–15.07)		
Superior temporal gyrus	Right	6.57 ± 1.16	6.52 (3.43–8.45)	1021.500	0.115
	Left	6.97 ± 1.25	6.93 (3.64–9.9)		
Middle temporal gyrus	Right	13.44 ± 6.87	12.71(8.67–58.73)	1030.500	0.130
	Left	12.03 ± 2.44	11.49 (5.56–17.33)		
Inferior temporal gyrus	Right	9.61 ± 2.37	9.7 (1.18–13.97)	1007.000	0.094
	Left	10.33 ± 2.45	10.6 (1.31–15.06)		

SD: Standard deviation; M: median; p: Mann–Whitney U test;

* statistical significance at p < 0.05.

**Table 7:** Comparison of the volumes of the lobus frontalis, lobus parietalis, and lobus temporalis in the right and left hemispheres of the control group.

Variables	Groups	Mean ± SD	M (range)	Test	p
Superior frontal gyrus	Right	14.39 ± 2.48	13.99 (9.91–21.36)	1123.500	0.383
	Left	14.06 ± 2.45	13.62 (9.13–20.13)		
Superior frontal gyrus medial segment	Right	6.37 ± 1.28	6.11 (3.96–9.26)	1034.500	0.137
	Left	6.01 ± 1.23	5.86 (2.79–9.16)		
Opercular inferior frontal gyrus	Right	3.4 ± 0.61	3.44 (2.3–5.05)	679.000	0.001*
	Left	2.88 ± 0.59	2.78 (1.75–4.26)		
Triangular inferior frontal gyrus	Right	2.9 ± 0.67	2.89 (1.61–4.6)	938.500	0.032*
	Left	3.21 ± 0.72	3.16 (1.93–5.02)		
Orbital inferior frontal gyrus	Right	1.02 ± 0.37	1.03 (0.26–2.01)	1070.500	0.216
	Left	1.11 ± 0.34	1.08 (0.4–1.98)		
Precentral gyrus	Right	12.15 ± 1.9	11.82 (7.55–17.14)	1014.500	0.104
	Left	12.54 ± 2.55	12.44 (1.09–18.25)		
Postcentral gyrus	Right	8.93 ± 1.31	8.76 (6.55–12.27)	620.000	0.001*
	Left	10.15 ± 1.35	10.02 (7.41–13.78)		
Postcentral gyrus medial segment	Right	1.01 ± 0.23	0.99 (0.56–1.59)	1112.500	0.343
	Left	0.96 ± 0.25	0.99 (0.46–1.5)		
Superior parietal lobule	Right	10.68 ± 1.63	10.58 (7.33–14.19)	1086.000	0.258
	Left	11.05 ± 1.62	11.02 (8.29–15.24)		
Precuneus	Right	12.14 ± 2.51	12.01 (8.08–19.42)	1218.000	0.825
	Left	11.99 ± 2.26	11.42 (8.35–17.84)		
Superior temporal gyrus	Right	7.12 ± 1.18	7.31 (4.32–9.39)	912.500	0.020*
	Left	7.65 ± 1.96	7.82 (0.81–11.85)		
Middle temporal gyrus	Right	13.39 ± 2.06	13.81 (10.81–18.86)	1040.000	0.148
	Left	12.56 ± 2.16	12.08 (9.31–18.1)		
Inferior temporal gyrus	Right	9.93 ± 1.8	9.77 (7.14–15.53)	817.000	0.003*
	Left	11.23 ± 2.15	10.75 (7.78–16.71)		

SD: Standard deviation; M: median; p: Mann–Whitney U test;

* statistical significance at p < 0.05.
